# Molecular Prevalence of *Babesia bigemina* and *Trypanosoma evansi* in Dairy Animals from Punjab, India, by Duplex PCR: A Step Forward to the Detection and Management of Concurrent Latent Infections

**DOI:** 10.1155/2013/893862

**Published:** 2013-08-29

**Authors:** Amrita Sharma, Lachhman Das Singla, Ashuma Tuli, Paramjit Kaur, Balwinder Kaur Batth, Mohammed Javed, Prayag Dutt Juyal

**Affiliations:** ^1^Department of Veterinary Parasitology, College of Veterinary Science, Guru Angad Dev Veterinary and Animal Sciences University, Ludhiana, Punjab 141004, India; ^2^Department of Mathematics, Statistics and Physics, Punjab Agricultural University, Ludhiana, Punjab 141004, India

## Abstract

Specific duplex polymerase chain reaction (PCR) was employed on 411 (386 cattle and 25 buffaloes) blood samples of dairy animals from 9 districts of Punjab, India, for simultaneous detection of *Babesia bigemina* and *Trypanosoma evansi*. The results were compared and correlated with conventional Giemsa stained thin blood smear (GSTBS) examination and haematological alterations to know the clinical status and pathogenicity of infections. The Bg3/Bg4 and TR3/TR4 primers were used in duplex PCR for *B. bigemina* and *T. evansi* amplified products of 689 bp and 257 bp, respectively. The overall prevalence by duplex PCR was found to be 36.49, 2.43, and 3.41% for *T. evansi*, *B. bigemina*, and dual infection, respectively. A more significant difference was observed for dual infection status (*P* ≤ 0.005) as compared to *T. evansi* (*P* ≤ 0.05) and *B. bigemina *(*P* ≤ 0.01) among various districts under study. A very low prevalence of *T. evansi* (0.73%) and *B. bigemina* (0.48%) was seen by GSTBS. The highly sensitive, specific, and cost-effective duplex PCR was able to detect latent *T. evansi* and *B. bigemina* infection in cattle and buffaloes. Haematological evaluation revealed marked pathology in *B. bigemina* infected group and in dual infected group in contrast to that infected with *T. evansi* alone.

## 1. Introduction

Babesiosis and trypanosomosis are two economically important vector-borne diseases of tropical and subtropical parts of the world including India. Bovine babesiosis caused by an apicomplexan haemoprotozoan parasite, *Babesia bigemina *(family Babesiidae, order Piroplasmida), is transmitted by brevirostrate tick, *Rhipicephalus *(*Boophilus*) *microplus,* causing significant morbidity and mortality in cattle and buffaloes. *Trypanosoma evansi,* the causative agent of the “Surra,” is mechanically transmitted by tabanid flies [1]. Dairy animals, especially bovines, which are bearing production stress along with other diseases, are potential viable host to these infections. Bovines act as reservoir hosts of surra as the course of disease remains subclinical. India suffers losses of about 57.2 million US dollars annually due to babesiosis in livestock [2]. For African trypanosomosis, estimated losses are to the tune of US $1.3 billion [3]; however, no data is available on the economic losses due to *T. evansi*.

The diagnosis of *T. evansi* and *B. bigemina* is routinely done by conventional parasitological techniques like Giemsa stained thin blood smear (GSTBS). Surra is characterized by fluctuating parasitaemia with periods of paroxysms and intermissions [4]. Giemsa stained blood smear examination is not a sensitive method to demonstrate parasites in the blood mainly because of the periodically cryptic nature of the parasite. The sensitivity of the technique may be enhanced by concentrating the blood (like buffy coat and minianion exchange chromatography) in place of using whole blood [5]. Despite the improvement in parasitological techniques, a significantly high proportion of infections remain undetected [6]. The recovered animals from *B. bigemina* infection may sustain subclinical infection or become carrier for other susceptible healthy animals in the herd and a source to infect the tick vectors. The serological tests, including the indirect fluorescent antibody test (IFAT) and the enzyme-linked immunosorbent assay (ELISA), are capable of detecting antibodies in carrier animals; therefore they are often used for monitoring surveillance and export certification [7]. Drawback of serological tests is that antibodies can be detected even years after recovery of infection though no active infection is prevalent, so these methods cannot help in revealing the exact picture of prevalence of infection at that particular point. For the diagnosis of subclinical and latent infection, nucleic acid based detection techniques like polymerase chain reaction (PCR) assay permit the identification of parasite at levels far below those identified by the commonly used conventional parasitological techniques for *T. evansi *[8] and *B. bigemina* [9]. Further, the detection of both of these haemoprotozoans simultaneously in a single reaction by duplex PCR will be both time and cost effective.

As both *B. bigemina *and* T. evansi *infections have almost similar types of signs and arthropod vectors of these two diseases also exist together in suitable tropical climatic conditions, it becomes necessary that an economical, specific, and sensitive technique be standardized and employed for simultaneous detection of these two diseases in suspected animals. Hence, the purpose of the present study was to standardize and employ a duplex PCR assay for simultaneous detection of clinical, subclinical, and latent carrier forms of* T. evansi *and* B. bigemina *infections, for knowing the prevalence of these infections in different districts of Punjab state and to further evaluate the haematological parameters to know the factors contributing to pathogenicity and to prevalence of these infections in dairy animals for their proper treatment and management. 

## 2. Materials and Methods

### 2.1. Sample Collection

Blood samples were randomly collected from 411 dairy animals (386 cattle and 25 buffaloes) during the period from May to October 2011 from 9 districts of Punjab state. Five mL of blood samples was collected in EDTA coated vacutainers from the jugular vein of the animals for DNA isolation and hematological parameter evaluation. Samples were brought from the field to laboratory in a thermos containing ice cubes and processed for haematology without delay. The samples were stored at −20°C till isolation of DNA.

### 2.2. Conventional Parasitological Method

Thin blood smears were prepared immediately after each blood collection. The blood smears were air-dried, fixed in methanol, stained with Giemsa, and examined microscopically for the presence of *B. bigemina* and *T. evansi*. Morphometric measurement of these haemoprotozoans was done by software DPZ-BSW (OLYMPUS) for parasitological confirmation of the species.

### 2.3. Molecular Diagnostic Method

#### 2.3.1. DNA Template Preparation

Genomic DNA was extracted from the whole blood collected in EDTA-coated vacutainers using HiMedia HiPurA Blood Genomic DNA MiniPrep Purification Spin Kit as per the given protocol (HiMedia Laboratories, India). The blood leucocytes from a three-day-old neonatal calf were included as negative control. *Trypanosoma evansi *parasites of cattle strain propagated in mice and purified by DEAE cellulose columns chromatography were used as *T. evansi *positive control, while *B. bigemina *parasites isolated from infected erythrocytes of clinically infected cattle were used as positive control for* B. bigemina.*

#### 2.3.2. Oligonucleotide Primers for Duplex-PCR

Oligonucleotide primers used for the establishment of duplex-PCR for *T. evansi* and *B. bigemina * targeted repetitive nucleotide sequences and small subunit ribosomal RNA sequence, respectively, as mentioned in Table 1.

#### 2.3.3. Generation and Visualization of Duplex-PCR Amplification

A total of 25 *μ*L PCR reaction mixture was constituted with KAPA 2G fast hot start ready mix (1X containing KAPA2G fast hot start DNA polymerase, KAPA 2G fast hot start PCR buffer, 0.2 mM dNTP each, and 1.5 mM MgCl_2_), with 15 pmol of Bg3/Bg4 primers and 12.5 pmol of TR3/TR4 primers and additional 1 mM MgCl_2_. The reaction was set in automated thermocycler with the following programme: initial denaturation at 95°C (5 min), 30 cycles of denaturation at 95°C (30 sec), annealing at 57°C (1 min), and extension at 72°C (1.5 min) with final extension at 72°C for 10 min. The amplified PCR products were separated by electrophoresis on 1% agarose gel and visualized under UV transilluminator for detection of 257 bp and 689 bp amplified product. 

#### 2.3.4. Specificity of Primers

Primers were examined for their specificity for each individual parasite species by amplification of DNA samples of individual parasite species as well as from the mixture of DNA samples derived from parasitologically positive samples of respective haemoprotozoan, with each set of primers. DNA extracted from cattle strain of *T. evansi* was propagated in mice and purified by DEAE cellulose columns chromatography as mentioned above. DNA extracted from isolated *Babesia bigemina *parasites frominfected erythrocytes from clinically infected cattle used as positive control for *B. bigemina*. Bovine leucocytes' DNA from a three-day-old neonatal calf was used as negative control. DNA isolated from erythrocytes of cattle infected with *Anaplasma marginale* and *Theileria annulata* was further used to check the specificity of the primers.

### 2.4. Haematological Analysis

The haematology of the whole blood was done with fully automated analyzed ADVIA 2120 haematology system (Siemens Health Care Diagnostic Inc., Deerfield, IL, USA) as per the instructions of the manufacturer. 

### 2.5. Statistical Analysis

Chi-square test was employed to compare prevalences of *T. evansi, B. bigemina, *and dual infections diagnosed by conventional parasitological and molecular techniques among various districts of Punjab. One-way analysis of variance (ANOVA) was applied on various hematological parameters to determine the variance in the animals of different groups, that is, group A (infected with *T. evansi *alone), group B (infected with* B. bigemina *alone), and group C (infected with both *T. evansi *and* B. bigemina*), as compared to the non-infected control animals (*P* < 0.05). Animals free from external parasites (ticks), negative for haemoprotozoans, having hematological parameters within normal range, free from any clinical sign, and with no history of any treatment given in the past were kept under noninfected control group. All the values are expressed as mean ± standard deviation.

## 3. Results

### 3.1. Specificity of PCR Primers

**Figure 1:**
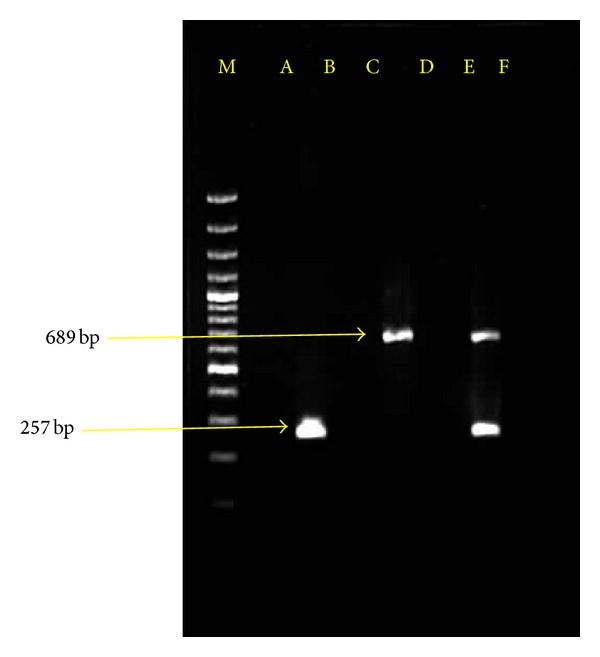
Agarose gel (1.5%) eletrophoresis showing amplified DNA from *B. bigemina* (689 bp) and *T. evansi* (257 bp). Lane M molecular size marker 100 bp plus. Lane A showing no amplification for *Theileria annulata* genomic DNA. Lane B showing amplification for *Trypanosoma evansi* genomic DNA. Lane C showing no amplification for host leucocyte DNA. Lane D showing amplification for *Babesia bigemina* genomic DNA. Lane E showing no amplification for *Anaplasma marginale *genomic DNA. Lane F showing amplification for *Babesia bigemina* and *Trypanosoma evansi *genomic DNA.

PCR amplification employed on each individual DNA sample (*T. evansi* and *B. bigemina*) using their specific primers led to the detection of expected fragments of size 257 and 689 bp, respectively. Each set of the primers was found to be specific for the respective parasite DNA, and amplification of nontarget DNA samples did not lead to the production of PCR products when other haemoparasite samples were used. Primer specific for one parasite species did not produce PCR products from any of the other parasite species (Figure 1). Parasite isolates from different districts were found to show uniform PCR amplification as all the isolates were amplified with the same set of primers used.

### 3.2. Relative Efficacy of Conventional Parasitological Method and Duplex PCR Assay and Corresponding Clinical Picture

Parasitaemia was observed only in 3 cases for *T. evansi* and in 2 cases for *B. bigemina* piroplasms by GSTBS. Common clinical manifestations of fever, pale mucous membrane, lacrimation, depression, and anorexia were observed in parasitologically positive animals for both types of infections. From the clinically positive cases of trypanosomosis, only one cattle was having corneal opacity and intermittent fever. Two cows positive for babesiosis showed history of haemoglobinuria and fever. Overall, it revealed the prevalence of *T. evansi *and *B. bigemina* to be only 0.73 and 0.48%, respectively, in nine districts of Punjab under study (Table 2). The highest prevalence of *T. evansi* was observed in district Jalandhar (7.5%) and that of *B. bigemina* was observed in district Ludhiana (2.06%). No case of coinfection of the two parasites was revealed by this method. 

Out of the total sample size, 35.5% of animals portrayed consistent signs of anemia, general weakness, history of reduced feed conversion, and occasional episodes of fever. Ticks were observed on 78.6% of the affected cattle and 9.4% of the affected buffaloes. 

**Figure 2:**
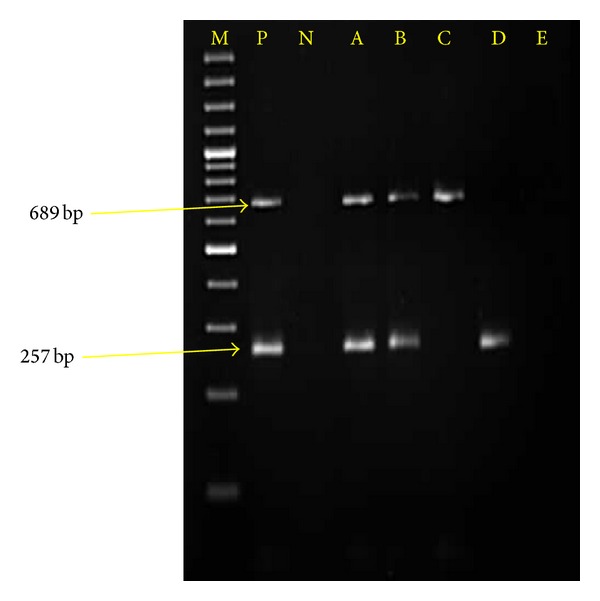
Agarose gel (1.5%) eletrophoresis showing amplified DNA from *B. bigemina* (689 bp) and *T. evansi* (257 bp). Lane M molecular size marker 100 bp plus. Lane P positive control. Lane N negative controls (genomic DNA from host leucocytes). Lanes A and B showing simultaneous amplified *B. bigemina* and *T. evansi* genomic DNA from the blood of animals positive for infection. Lane C showing amplified *B. bigemina* genomic DNA from the blood of animal infected for *B. bigemina* alone. Lane D showing amplified *T. evansi* genomic DNA from the blood of animal infected for *T. evansi* alone. Lane E showing no amplification indicating being negative for dual infection by PCR.

Out of 411 samples examined, 3.41% were found to have concurrent infection of both protozoans when targeted for duplex PCR (Figure 2). This PCR based diagnosis (Figure 2) revealed an overall prevalence of *T. evansi* to be 36.49% and that of *B. bigemina* to be only 2.43%. A more significant difference was observed for dual infection status (*P* ≤ 0.005) as compared to *T. evansi *(*P* ≤ 0.05) and* B. bigemina *(*P* ≤ 0.01) among various districts under study, with highest prevalence of coprevalence of both protozoa in district Jalandhar (7.5%), while that of *T. evansi* and *B. bigemina* alone in district Sangrur (47.82%) and Mansa (7.41%), respectively (Table 2). Based on the prevalence of the diseases, the veterinarians of the particular regions were asked to treat the animals, specifically. Population of the ticks was mainly constituted by *R.* (*Boophilus) microplus* followed by * Hyalomma anatolicum anatolicum. * Fourteen *Tabanus *spp. flies could be collected only from Jalandhar, Ludhiana, and Patiala districts.

 PCR assay could effectively diagnose trypanosome infection in animals under study revealing 41.33% of subclinical cases (with clinical signs but negative by GSTBS) and 56.6% of carrier cases (GSTBS negative and with no clinical signs) that harboured infection (Table 3). Correspondingly similar PCR assay report was observed in case of *B. bigemina* infection which diagnosed 12.5% clinical, 18.75% subclinical, and 68.7% carrier cases. 

### 3.3. Species-Wise Comparative Prevalence in Cattle and Buffaloes

Among cattle, the prevalence of 36.01, 2.07, and 2.84% was observed for infections of *T. evansi, B. bigemina *and concurrent infection of both parasites, respectively, by duplex PCR method of diagnosis. Prevalence of these infections was more in buffaloes being 44.00, 8.00, and 12.00% for *T. evansi, B. bigemina, *and concurrent infection of both parasites (Table 2). The coprevalence of both haemoprotozoans was found to be higher in buffaloes (12%) as compared to cattle (2.84%) by duplex PCR. 

### 3.4. Correlation of the Infection Status with Haematological Alterations

**Figure 3:**
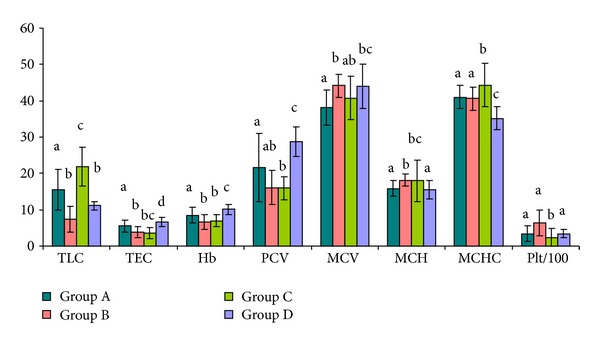
Haematological parameters of animals infected with *T. evansi* (group a), *B. bigemina *(group b), both *T. evansi* and *B. bigemina* (group c), and non infected control (group d). Values of TLC, TEC, Hb, PCV, MCV, MCH, MCHC, and Plt with different letters a–d differ significantly at *P* ≤ 0.05.

Leucogram (Table 4, Figure 3) showed significant (*P* < 0.001) leucocytosis in *T. evansi* infected animals of group A. However, in group B (*B. bigemina* infected animals) neither leucocytosis nor leucopenia was seen as compared to healthy animals of group D, while animals of group A and group C showed significant leucocytosis as compared to the healthy controls.

The total erythrocyte count (TEC) was found reduced in infected groups as compared to healthy animals (Table 4). Haemoglobin (Hb) was significantly lower (*P* < 0.05) in all infected groups as compared to healthy controls. In *B. bigemina* infected and dual infected animals, Hb was decreased more significantly. Packed cell volume (PCV) was significantly lower in all the infected groups as compared to healthy control animals, the decrease being significant in group C animals as compared to group A animals. Mean corpuscular volume (MCV) was significantly lower in *T. evansi* infected animals as compared to *B. bigemina* infected animals. Mean corpuscular haemoglobin (MCH) decreased significantly in *B. bigemina *and dual infected group as compared to *T. evansi* infected animals and healthy control animals. Mean corpuscular haemoglobin concentration (MCHC) was on higher side in infected groups as compared to healthy control group, with the increase being significantly higher in dual infected groups as compared to all other groups. Thrombocytosis was observed in *B. bigemina* infected group as compared to *T. evansi* infected or dual infected groups (Table 4).

Overall, microcytic normochromic type of anemia was observed in all the three infected groups due to significant reduction (*P* < 0.01) in the mean corpuscular volume (MCV). 

## 4. Discussion

The morphometric measurement of *T. evansi* was 25.8 ± 0.8 *μ*m and that of *B. bigemina* was 2.9 ± 0.2 *μ*m in GSTBS. These measurements were in accordance with the standard dimensions described for *T. evansi *and *B. bigemina*. PCR based diagnosis showed the prevalence of *T. evansi* and *B. bigemina* to be 36.49 and 2.43%, respectively, as compared to much lower prevalences (*T. evansi* 0.73% and *B. bigemina* 0.48%) observed by GSTBS. It justifies the sensitivity of duplex PCR as it was able to expose the subclinical and carrier cases of both *T. evansi* and *B. bigemina* which would have led to further attention for treatment and control in lieu being obscured. This very well corroborates with the fact that due to antigenic variations, cryptic infections are seen in *T. evansi *leading to immunosuppression and nondetectable parasitaemia during the infection processes [4, 12, 13]. A higher subclinical incidence of *T. evansi* (34.6%) than *T. annulata* (16%) and *B. bigemina* (0.6%) in cattle has been reported in Karnataka (India) [14] by conventional single-plex PCR. Exploring of the carrier and subclinical cases by more sensitive techniques, namely, PCR, will be helpful in judging the potential threat of the diseases in a particular area or farm. This will further help in the quick and accurate treatment strategies and management of the diseases [15] as was advised in the present study.

 Epidemiological findings [16] have revealed *R. microplus* as the predominant tick population (85.28%) in Punjab (India) which is responsible for transmission of *B. bigemina* in cattle and buffaloes. By using GSTBS as well as PCR, a higher number of cases of haemoprotozoa infection were observed in buffaloes than in cattle. This may be due to great variation in the sample size of cattle and buffaloes [17]. Previously, parasitological prevalences by GSTBS examination were reported to be 7.92% for *T. evansi* [18] and 5.94% for *B. bigemina *in dairy animals [19] in Punjab state. History of presence of tabanid fly in the vicinity was frequently recorded from the area of Moga, Ferozepur, and Sangrur districts. Farms at district Jalandhar were in the vicinity of paddy fields which are conducive for breeding of tabanid flies. This may be attributed to high prevalence of *T. evansi* in Jalandhar district.

 No observation of mixed infection of* T. evansi *and* B. bigemina* by GSTBS examination may be due to low sensitivity of this conventional parasitological technique [15]. Buffaloes are considered to be more resistant towards the haemoprotozoan diseases [20]. Hence buffaloes act as reservoir hosts to other animals and may become clinically ill under stress conditions. Further, it was observed that crossbred cattle are more sensitive to these haemoprotozoan infections as compared to buffaloes as the clinical cases were encountered only in crossbred cattle. Infection rate of *T. evansi* and *B. bovis* has been reported to be 13.7 and 7.4% in buffaloes as depicted by duplex PCR [21]. A very little or no mixed infection was observed in dairy cows; it was 3.7 and 0%, respectively, for *T. evansi* and *B. bovis*.

Duplex PCR, due to its high sensitivity and specificity, is able to detect low level of infections and can be suitable for epidemiological study of *T. evansi* and *B. bigemina* infection in cattle and buffaloes. The incidence of coinfection of both protozoa is lesser which may be due to different vectors responsible for their transmission. The reason for higher molecular prevalence of *T. evansi* as compared to *B. bigemina* can be corroborated with the fact that the tabanid flies have interrupted feeding habit and can travel longer distances as compared to the ticks.

The observed leucocytosis in animals of groups C and A is in agreement with some previous reports [23–25]. It may be due to the multiplicity of the antigens resulting into more stimulation of immune system. The insignificant changes in total leucocyte count in *B. bigemina* infected group may be due to the breakdown of red blood cells by the protozoan stimulating phagocytic cells such as lymphocytes and monocytes to clean up the toxic remnants of ruptured red blood cell [26]. The significant decrease in Hb, TEC, and PCV in *B. bigemina* infected group as compared to other groups may be due to intravascular haemolysis, which is supported by the previous reports [27]. Overall hematological revelations showed that anemia was more severe in *B. bigemina* infected and dual infected animals as compared to the animals infected with *T. evansi *alone. Results indicated that even subclinical and latent carrier infections diagnosed by molecular means are responsible for inducing pathogenicity.

## 5. Conclusion

The present study reveals the concurrent latent infection of babesiosis and trypanosomosis in cattle and buffaloes in Punjab. The diagnosis of the dual latent infections by duplex PCR will be helpful in control and prevention of these deadly diseases as this technique is both time and cost effective. The technique is useful in the detection of molecular prevalence of the haemoprotozoan diseases. The haematological analysis supported the pathogenicity of the dual latent infection. 

## Figures and Tables

**Table 1:** Oligonucleotide primers used for establishment of duplex PCR.

Haemoprotozoan	Primer	Region amplified	Product size
*B. bigemina* [10]	*Bg3 5*′*TAG TTG TAT TTC AGC CTC GCG 3*′	Small subunit ribosomal RNA sequence of *B. bigemina *	689 bp
	*Bg4 5*′* AAC ATC CAA GCA GCT AHT TTA G 3*′		
*T. evansi* [11]	*TR3 5*′* GCG CGG ATT CTT TGC AGA CGA 3*′	Repetitive nucleotide sequences of *T. evansi *	257 bp
	*TR4 5*′* TGC AGA CAC TGG AAT GTT ACT 3*′		

**Table 2:** Prevalence of *B. bigemina* and *T. evansi* alone and in combination by Giemsa stained thin blood smear (GSTBS) examination and duplex polymerase chain reaction (PCR).

Districts covered	Samples tested	Positive for *T. evansi* alone		Positive for *B. bigemina *		Positive for both *T. evansi* and *B. bigemina *	
		By GSTBS	By PCR	By GSTBS	By PCR	By GSTBS	By PCR
Ferozepur	25	0	0	0	0	0	0
Mansa	54	0	20 (37.03%)	0	4 (7.41%)	0	1 (1.85%)
Moga	18	0	6 (33.33%)	0	0	0	0
Sangrur	23	0	11 (47.82%)	0	1 (4.34%)	0	1 (4.34%)
Bathinda	66	0	23 (34.84%)	0	0	0	0
Fatehgarh Sahib	54	0	22 (40.74%)	0	1 (1.85%)	0	0
Patiala	34	0	15 (44.11%)	0	0	0	2 (5.88)
Jalandhar	40	3 (7.5%)	12 (30%)	0	0	0	3 (7.5%)
Ludhiana	97	0	41 (42.26%)	2 (2.06%)	4 (4.12%)	0	7 (7.22%)

Total	411	3 (0.73%)	150 (36.49%)	2 (0.48%)	10 (2.43%)	0	14 (3.41%)
*χ* ^2^		24*	67.2*	16**	20.6***	NS	27.14*
							
Cow	386	3 (0.77%)	139 (36.01%)	1 (0.25%)	8 (2.07%)	0	11 (2.84%)
Buffalo	25	0	11 (44%)	1 (4%)	2 (8%)	0	3 (12%)
*χ* ^2^		NS	109.22*	NS	NS	NS	4.57**

*P ≤ 0.005, **P ≤ 0.05, and ***P ≤ 0.01.

**Table 3:** Correlation of infection of *T. evansi*, *B. bigemina*, and dual infection with sensitivity of PCR.

	*T. evansi* positive (150)			*B. bigemina* positive (10)			Mixed infection positive (14)		
	+/CS	−/CS	−/NCS	+/CS	−/CS	−/NCS	+/CS	−/CS	−/NCS
PCR positive	3 (2%)	62 (41.33)	85 (56.6)	2 (20.0)	3 (30)	5 (50)	0	9 (64.85)	5 (35.71)

+/CS: sample positive by slide and animal showed clinical signs (clinical cases).

−/CS: sample negative by slide and animal showed clinical signs (subclinical cases).

−/NCS: sample negative by slide and animal showed no clinical signs (latent carriers).

**Table 4:** Haematological values of animals infected with *B. bigemina, T. evansi*, and dual infection.

	Parameter	TLC (×10^3^ cells/*µ*l)	TEC (×10^6^ cells/*µ*l)	Hb (g/dL)	PCV (%)	MCV (fL)	MCH pg	MCHC g/dl	Plt (×10^3^ cells/*µ*l)
Positive for *T. evansi* alone (group A)	Total samples tested = 92	15.39^a^ ± 5.59	5.50^a^ ± 1.63	8.51^a^ ± 2.14	21.61^a^ ± 9.28	38.12^a^ ± 4.92	15.81^a^ ± 2.17	41.02^a^ ± 3.20	341.51^a^ ± 214.97
Positive for *B. bigemina* alone (group B)	Total samples tested = 5	7.376^b^ ± 3.61	3.846^b^ ± 1.44	6.60^b^ ± 1.95	16.10^ab^ ± 4.69	44.16^b^ ± 3.24	18.16^b^ ± 1.71	40.58^a^ ± 3.12	632.00^b^ ± 348.59
Positive for * T. evansi* and *B. bigemina* (group C)	Total samples tested = 8	21.87^c^ ± 5.439	3.59^bc^ ± 1.43	6.96^b^ ± 1.62	15.91^b^ ± 3.18	40.75^ab^ ± 6.03	18.01^bc^ ± 5.73	44.28^b^ ± 5.96	240.87^a^ ± 233.59
Non infected control group (group D)	Total number of samples = 30	11.06^b^ ± 1.25	6.68^d^ ± 1.29	10.09^c^ ± 1.39	28.76^c^ ± 4.12	43.99^bc^ ± 6.04	15.42^a^ ± 2.55	35.12^c^ ± 3.18	336.83^a^ ± 112.62
Normal range (NR) [22]		4–12	5–10	8–15	24–46	40–60	14.4–18.6	26–34	100–800

Values of TLC, TEC, Hb, PCV, MCV, MCH, MCHC, and Plt with different superscripts a–d differ significantly at *P* ≤ 0.05 through the columns.

TLC: total leucocyte count, TEC: total erythrocyte count, HB: haemoglobin, PCV: packed cell volume, MCV: mean corpuscular volume, MCH: mean corpuscular haemoglobin, MCHC: mean corpuscular haemoglobin concentration, and Plt: platelets.

## Abbreviations

PCR: Polymerase chain reaction
GSTBS: Giemsa stained thin blood smear.

## References

[1] Sumba AL, Mihok S, Oyieke FA (1998). Mechanical transmission of *Trypanosoma evansi* and *T. congolense* by *Stomoxys niger* and *S. taeniatus* in a laboratory mouse model. *Medical and Veterinary Entomology*.

[2] McLeod R, Kristjanson P (1999). *Final Report of Joint ESYS/International Livestock Research Institute/Australian Centre for International Agricultural Research Tick Cost Project-Economic Impact of Ticks and Tick-Borne Diseases to Livestock in Africa, Asia and Australia*.

[3] O.I.E. (2010). Trypanosoma evansi infections (surra). *Terrestrial Manual: Chapter 2.1.17. Office International Des Epizooties*.

[4] Gill BS (1991). *Trypanosomes and Trypanosomiases of Indian Livestock*.

[5] Reid SA, Husein A, Copeman DB (2001). Evaluation and improvement of parasitological tests for *Trypanosoma evansi* infection. *Veterinary Parasitology*.

[6] Luckins AG, Allen WR, Higgins AJ, Mayhew IG, Snow DH, Wade JF (1992). Protozoal diseases of camels. *Proceedings of the 1st International Conference*.

[7] Araújo FR, Madruga CR, Leal CRB (1998). Comparison between enzyme-linked immunosorbent assay, indirect fluorescent antibody and rapid conglutination tests in detecting antibodies against Babesia bovis. *Veterinary Parasitology*.

[8] Desquesnes M, Dávila AMR (2002). Applications of PCR-based tools for detection and identification of animal trypanosomes: a review and perspectives. *Veterinary Parasitology*.

[9] Figueroa JV, Chieves LP, Johnson GS, Buening GM (1993). Multiplex polymerase chain reaction based assay for the detection of *Babesia bigemina*, *Babesia bovis* and *Anaplasma marginale* DNA in bovine blood. *Veterinary Parasitology*.

[10] Ellis J, Hefford C, Baverstock PR, Dalrymple BP, Johnson AM (1992). Ribosomal DNA sequence comparison of Babesia and Theileria. *Molecular and Biochemical Parasitology*.

[11] Wuyts N, Chokesajjawatee N, Panyim S (1994). A simplified and highly sensitive detection of *Trypanosoma evansi* by DNA amplification. *The Southeast Asian Journal of Tropical Medicine and Public Health*.

[12] Singla LD, Sharma SK, Juyal PD (2008). Pharmacokinetics of diminazene in combination with procaine and antipyrine in *Trypanosoma evansi* infected buffalo calves. *Indian Journal of Animal Sciences*.

[13] Singla LD, Juyal PD, Sharma NS (2010). Immune responses to haemorrhagic septicaemia (HS) vaccination in *Trypanosoma evansi* infected buffalo-calves. *Tropical Animal Health and Production*.

[14] Nair AS, Ravindran R, Lakshmanan B (2011). Haemoprotozoa of cattle in Northern Kerala, India. *Tropical Biomedicine*.

[15] Juyal PD, Singla LD, Kaur P, Tandon V, Dhawan BN (2005). Management of surra due to *Trypanosoma evansi* in India: an overview. *Infectious Diseases of Domestic Animals and Zoonosis in India*.

[16] Haque M, Jyoti J, Singh NK, Rath SS, Ghosh S (2011). Epidemiology and seasonal dynamics of Ixodid ticks of dairy animals of Punjab state, India. *Indian Journal of Animal Sciences*.

[17] Yadav CL, Gupta RP, Ruprah NS (1985). The prevalence of haemoprotozoan infections in cattle and buffaloes. *Indian Veterinary Medical Journal*.

[18] Aulakh GS, Singla LD, Kaur P, Alka A (2005). Bovine babesiosis due to *Babesia bigemina*: haematobiochemical and therapeutic studies. *Indian Journal of Animal Sciences*.

[19] Aulakh GS, Singla LD, Singh J, Sobti RC, Sharma VL (2005). Bovine trypanosomosis due to *Trypanosoma evansi*: clinical, haematobiochemical and therapeutic studies. *New Horizons in Animal Sciences*.

[20] Singh DK, Dolan TT (September 1990). Recent developments. *The Research and Control of Theileria Annulata Proceedings of a Workshop Held at Ilrad*.

[21] Shi Y, Huang W, Zhang W (2010). Rapid and sensitive detection of *Trypanosoma evansi* and Babesia bovis in bovine by double PCR. *Guangxi Agricultural Sciences*.

[22] Kahn CM, Line S, Aiello SE (2005). *Reference Guide Table 6 in Merck Manual*.

[23] Walia PS, Kalra IS, Juyal PD, Ahuja SP (1996). Role of activity of *Trypanosoma evansi* in inducing anemia and immunomodulation in buffalo calves. *Journal of Veterinary Parasitology*.

[24] Kulkarni MD, Nisal MB, Rao MKJ, Kulkarni PE (1997). Haemodynamic studies of trypanosomiasis in buffalo (*bubalus bublais*). *Indian Veterinary Journal*.

[25] Kaur P, Juyal PD (2003). Haematobiochemical changes in experimental surra in cow calves treated with diminazene aceturate along with antipyrine and procaine. *Indian Veterinary Journal*.

[26] Hussein AH, Mohammed NAES, Mohammed HK Theileriosis and babesiosis in cattle: haemogram and some biochemical parameters.

[27] Sharma AK, Katoch RC, Nagal KB, Kishtwaria RS, Sharma SK (2000). Bovine Babesiosis in Palam Valley of Himachal Pradesh. *Indian Veterinary Journal*.

